# Prevention of parent to child transmission services and interventions - coverage and utilization: A cohort analysis in Gujarat, India

**DOI:** 10.4103/2589-0557.74983

**Published:** 2010

**Authors:** Urvish Joshi, Amimuddin Kadri, Sudeshna Bhojiya

**Affiliations:** Department of Community Medicine, Smt. NHL Municipal Medical College, Ahmedabad, Gujarat, India; 1 Department of Community Medicine, PDU Medical College, Rajkot, Gujarat, India; 2 Department of Community Medicine, Gujarat State AIDS Control Society, Ahmedabad, Gujarat, India

**Keywords:** HIV in exposed children, HIV testing of pregnant women, MB pair, prevention of mother to child transmission

## Abstract

**Background and Objectives:**

Risk of vertical transmission (largest source of HIV in children) reduces from 33% to 3% with effective prevention of mother to child transmission (PMTCT) interventions. NACP-III has got an objective of testing all pregnant women for earliest linkage with PMTCT. Study was carried out to find out PPTCT service coverage, dropouts, intervention efficacy with other determinants.

**Materials and Methods:**

At ICTCs, registered ANCs are counseled and tested for HIV. HIV +ve ANCs are additionally linked to services and followed-up for institutional delivery, sdNVP, nutrition and children testing. HIV +ve ANCs since 2005 subsequently delivered till June 2008 and their exposed children in Gujarat’s category A, B districts constituted study cohort.

**Results:**

259622 pregnant women registered, 72.1% were counseled pre-test, 83.4% of them tested, 74.4% received post-test counseling. 541 ANCs were detected HIV+ve. 45.5% delivered institutionally, 12.8% were unregistered. 12.1% were cesarian section and 66% delivered vaginally. 96.8% were live births, 92.13% mother-baby pair received sdNVP. 35% children could be traced till 18 months, 89% were alive. 90% were tested, 3 were found HIV +ve. Of them, none received MB Pair. Two were delivered vaginally, two received mixed feeding, two children’s mothers were not linked with ART.

**Conclusions:**

PMTCT services – counseling and testing should be provided to all ANCs. EDD-based tracking, institutional deliveries, postnatal counseling to be encouraged along with complete MB pair coverage, capacity building of concerned staff regarding delivery of HIV+ve ANCs and exposed children tracking.

## INTRODUCTION

Mother-to-child transmission (MTCT) is the largest source of HIV infection in children below the age of 15 years.[1] According to National AIDS Control Organization (NACO), about 30,000 infants are estimated to acquire HIV infection each year.[2]

Infection to newborn is transmitted by mother perinatal, however, considering the role of male partner in transmission of infection to woman, in India it is appropriately called parent-to-child transmission (PTCT).

Gujarat HIV Sentinel Surveillance (HSS 2007) covering 9517 pregnant women (ANCs), showed the median positivity rate of 0.46% HIV infection among ANCs.[3]

In absence of intervention, rate of vertical transmission is estimated to be around 30–33% which drops down to around 3% with effective antenatal, intranatal and postnatal PPTCT interventions. Intrapartum ante-retroviral treatment (ART) prophylaxis (Nevirapine -NVP), viral load-based ART to ANCs, mode of delivery and infant feeding are factors considered to be determinant for seropositivity in children born to these women (exposed children).[4]

National AIDS Control Programme - phase III (NACP III) has got a long-term objective of testing 22 million by 2012 inclusive of testing of all ANCs.[5]

Latter is being done with the purpose of putting seropositive ANCs on ART as soon as possible in order to derive its maximum benefit to prevent opportunistic infections (most importantly tuberculosis) and to provide single-dose NVP (sdNVP) as an ART regime to both mother and baby to prevent vertical transmission.

In Gujarat, ANC testing was started in 14 Integrated Counseling and Testing Centers (ICTCs) in 2005–’06 which is now scaled up to 400 centers including 289 stand-alone and 111 facility-integrated ICTCs (58 24 × 7 PHCs and 53 Public Private Partnerships). Estimated annual pregnancies for Gujarat are estimated to be around 14 lakh, of which 55% deliveries are taking place in public sector.[6] Strategy being followed in NACP-III is to make district, the unit of intervention.

Present study has been carried out with the following objectives:

To find out the coverage of PPTCT services and to assess the dropouts at several levels owing to its complex service delivery system To find out efficacy of these interventions in terms of reducing transmission rates in exposed children in accordance with other determinants’ seropositivity.

## MATERIALS AND METHODS

ANCs registering at various health facilities are given pre-test counseling at ICTCs in groups with an average duration between three women being 20–40 min average. With obtained verbal consent, they are tested for HIV by three rapid tests and results whether positive or negative are shared by the ICTC counselor along with individual post-test counseling.

While seronegative ANCs are counseled on HIV prevention and risk reduction behavior, HIV seropositive ANCs are additionally provided psychosocial support on disclosure issues and spousal testing, linkage to TB testing and ART services, importance of institutional delivery and intrapartum sdNVP, postpartum follow-up and infant feeding.

During the delivery of these seropositive ANCs, sdNVP regime of ARV (200 mg tablet) is given to the woman at the onset of labor and NVP syrup (2 mg/kg of body weight) is offered to the babies within 72 h of the birth.[7]

Exposed children are tested at completion of 18 months through HIV rapid tests for seropositivity confirmation.[4]

In Gujarat, 10 districts fall in category A and B as per the categorization (2006), based on results of surveillance for the last three years;[8] therefore, the seropositive ANCs detected since inception of services (2005) and subsequently delivered till June 2008 in these 10 districts constituted the cohort for study objectives. Babies born to them were followed up till 18 months for confirmation of their seropositivity status.

## RESULTS

**Figure 1:**
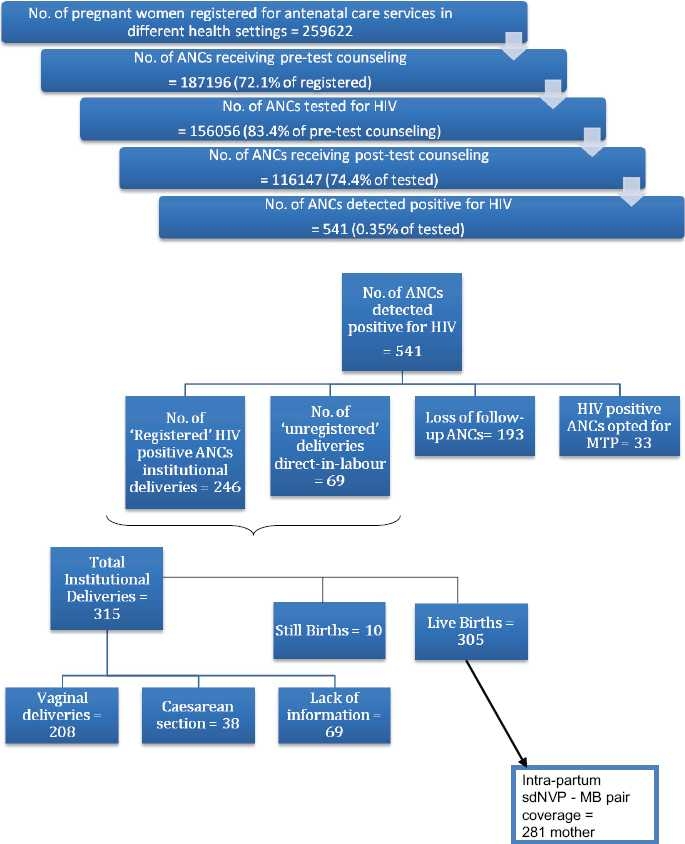
PPTCT - Counseling and testing services and cohort of follow-up of the HIV positive ANCs

Since 2005 till June 2008, in 10 category A and B districts of the state, 72.1% ANCs out of all registered were counseled before HIV testing; out of them, 83.4% were tested for HIV. 74.4% such tested women were given post-test counseling before leaving the health setting including all seropositive pregnant women [Figure 1]. Total 541 ANCs were detected positive for HIV, sero-prevalence being 0.35% and were further tracked down as per Figure 1.

Gap between ANC registrations versus receipt of pre-test counseling was significant in some districts, service uptake being 54.3% (Vadodara), 68.6% (Rajkot), 72.3% (Surat) and 72.5% (Surendranagar). In Rajkot, 57.5% ANCs counseled pre-test were actually tested for HIV. Post-test counseling was not given to all tested pregnant women (74.4%) in these districts, significant districts being Surat (59.4%), Bhavnagar (68.9%) and Vadodara (70.9%) in terms of service uptake [Table 1].

**Table 1:** Counseling and testing services under PPTCT and seropositivity detection

District	Cat.*	Registered ANCs[1]	ANCs received pre-test counseling[2] (% of 1)	ANCs tested for HIV[3] (% of 2)	ANCs received post-test counseling[4] (% of 3)
Banaskantha	A	5978	4690 (78.5)	3997 (85.2)	3558 (89.0)
Dahod	A	4821	3937 (81.7)	3768 (95.7)	2851 (75.7)
Mehsana	A	6186	5851 (94.6)	5392 (92.2)	4465 (82.8)
Navsari	A	8429	7530 (89.3)	6848 (90.9)	5986 (87.4)
Surat	A	62879	45484 (72.3)	33504 (73.7)	19897 (59.4)
Surendranagar	A	10746	7786 (72.5)	6605 (84.8)	4595 (69.6)
Ahmedabad	B	71683	55230 (77.0)	53854 (97.5)	42325 (78.6)
Bhavnagar	B	14181	10797 (76.1)	9098 (84.3)	6264 (68.9)
Rajkot	B	37254	25554 (68.6)	14683 (57.5)	13226 (90.1)
Vadodara	B	37465	20337 (54.3)	18307 (90.0)	12980 (70.9)
Total		259622	187196 (72.1)	156056 (83.4)	116147 (74.4)

* Categorization of districts[8]

**Figure 2:**
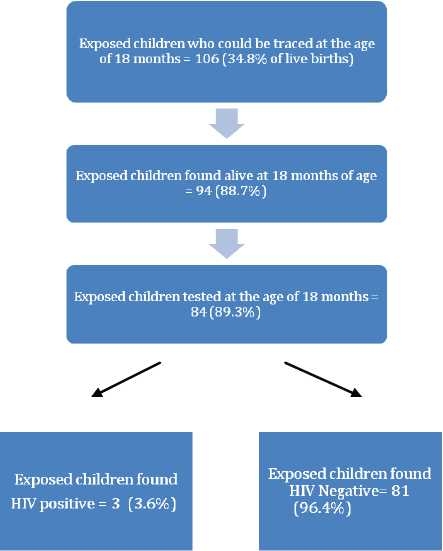
Follow-up of the cohort of exposed children (Children born to HIV positive mothers = exposed children)

106 out of 305 live births – exposed children could be traced till the age of 18 months (34.8%). Tracing followed as per Figure 2.

Among these exposed children tested for HIV, 9 children did not receive sdNVP-MB pair, out of which three were tested seropositive. Out of 49 vaginal deliveries, 2 children were tested seropositive, while 1 out of 35 cesarean sections turned out to be positive. No seropositivity was reported among exposed children on exclusive replacement feeding while 1 child out of 42 on exclusive breastfeeding and 2 out of 8 children on mixed feeding were reported seropositive. One out of 5 children was reported to be seropositive whose mother was receiving ART during pregnancy at the time of delivery, while 2 out of 15 children whose mothers were recommended ART during pregnancy but were not on ART, reported to be seropositive [Table 2].

**Table 2:** Seropositivity status of exposed children at 18 months in association with sdNVP prophylaxis, mode of delivery, breastfeeding and ART to the ANC

Factors			HIV seropositivity status in exposed children	
			Total	HIV seropositive
SdNVP (MB pair)		Received	75	0 (0.0)
		Not received	9	3 (33.3)
Total			84	3 (3.6)
Mode of delivery		Cesarean section	35	1 (2.9)
		Vaginal delivery	49	2 (4.1)
Total			84	3 (3.6)
History of breastfeeding		Exclusive breastfeeding	42	1 (2.4)
		Exclusive replacement feeding	34	0 (0.0)
		Mixed feeding	8	2 (25.0)
Total			84	3 (3.6)
Mother receiving ART during the time of delivery	ART recommended	Mother on ART	5	1 (20.0)
		Mother not on ART	15	2 (13.3)
	ART not recommended		64	0 (0.0)
Total			84	3 (3.6)

Values in parenthesis are in percentage

## DISCUSSION

PPTCT services in Gujarat started in the form of ICTCs since 2005. Initially, the services were available at medical colleges and district-level health facilities; within two years, there was a steady upscale of the service delivery model. There has been a massive increase in number of ICTCs covering all CHCs of category A and B districts. This makes the program unique in the sense that it offers the services package to all those women who access the government hospitals at the block level. At the same time, mechanisms involved are complex liasioning amongst the key service providers like the counselors, medical officers, staff nurses, laboratory technicians and ORWs who individually come in contact with the ANCs at different phase of service delivery.

Despite having similar service delivery model in all 10 districts, inter-district variations were found in terms of dropout of ANCs at various stages of counseling and testing services.

ICTCs are considered to be the entry point for ANCs to the healthcare system, and HIV seroprevalence in ANCs represents the severity of epidemic in general population, hence all ANCs should be tested for HIV. In districts like Vadodara, Rajkot, Surat and Surendranagar, huge gaps were reported among ANCs registering at the health facilities and those receiving pre-test counseling at ICTCs. These are the districts with high industrial development, labor migration, and existence of surrounding tribal areas. Linkage of all registered ANCs to the ICT services thus is imperative by close district-specific monitoring.

In Rajkot, only half of the ANCs counseled pre-test were actually tested for HIV (57.5%) suggesting the imperativeness of quality of counseling, referral systems, ready availability of testing facilities and simple and quick reporting channels apart from strengthening the internal coordination within the health facility, although these figures are significantly higher than those reported in rural Maharashtra (13.3%) in the study carried out by Sinha G *et al*.[9]

Post-test counseling is considered to have an essential role in bondage, subsequent follow-up and finally institutional delivery of ANC within the health setting; quality of same mainly depends upon skills and motivational levels of ICTC counselor. Cumulatively for all 10 districts, it was provided to only 3/4th of the pregnant women (74.4%) tested for HIV and districts with problems were mainly Surat, Bhavnagar and Vadodara.

Thus, not even 2/3rd of all registered ANCs (60.1%) were actually tested for HIV and only about half of all registered ANCs (44.7%) actually received exclusive ICT services. These variations and gaps in various ANC dropouts can be taken into considerations for subsequent district monitoring and evaluation.

HIV is believed to be concentrated in urban areas and earlier the services also focused only the urban areas, hence the seropositivity happened to be 0.46% of total testing which has come down gradually year by year and corroborate with the subsequent findings of HIV sentinel surveillance in the state. The overall HIV positive rate among those tested was 1.67% in the study carried out by Dandona L *et al*, in 16 sampled PPTCT centers of Andhra Pradesh, India in 2005–’06. Gujarat falls in the category of state with moderate prevalence and hence the low sero-positivity compared to Andhra Pradesh which falls in high-risk category.[10]

Out of all 541 detected seropositive ANCs, 12.8% ANC were detected only at the time of delivery (direct-in-labor cases), which shows the immense need to test not only the registered ANCs but also the unregistered emergency cases before delivery in order to provide prophylactic sdNVP in due time. This also points at the need to identify and plug the gaps in the RCH and other government health programs so that detection and linkage to care, support and treatment can be done earlier.

While 6.1% seropositive women opted to terminate pregnancy in first trimester, more than 1/3 ^rd^ of seropositive ANCs (35.7%) were not reported delivering institutionally, which may be a case of delivering domestically or in private sector or lost to follow-up. Institutional delivery is the mainstay of PPTCT services especially in context of intrapartum sdNVP interventions and further postpartum counseling. Such gap would contribute to sustainment as well as increase in HIV seropositivity in the society.

Vaginal was the predominant mode among all reported institutional deliveries (66%), while information regarding mode of delivery was not available in case of 22% ANCs. Elective cesarean is the preferred mode of delivery in seropositive ANCs, which again is possible only by effective antepartum follow-up.[11] Predominance of vaginal deliveries silently suggests the need for sensitization of involved staff.

Simplicity, low-cost and efficacy are the reasons of the sdNVP prophylaxis to mother and infant as to why it is being widely used in resource-constrained settings for PMTCT programs globally as well as in India.[7,11] Coverage of sdNVP to both mother and baby was reported in 92.13% of the live births which ideally should have been 100%. Nevirapine only to mother or only to baby is considered to be resulting into decreased efficacy of intervention. MB pair coverage thus was found to be only about half (51.9%) against detected seropositive ANCs, which highlight the immense need and channelized efforts to improve institutional deliveries of seropositive ANCs.

Maternal antibodies are expected to be present in the baby at least till the age of 18 months and hence rapid tests detecting antibodies are designated for 18 months.[12] The follow-up of babies after their birth in order to get them tested at 18 months is a difficult task in the rural settings considering migration and other social factors. Just like ANCs, drop-out in service utilization was observed among exposed children also. Only about 1/3^rd^ of the exposed children (34.8%) could be traced till the age of 18 months which again shows the weak follow-up. 11.3% children had died by then. 10 children were traced successfully but could not be tested for various reasons which again shows a worrisome dropout.

HIV seropositivity among the tested children was found to be 3.6% and none of the three seropositive children had received sdNVP-MB pair. Srijayanth Parameshwari *et al*, in their study in Tamil Nadu reported two infants turning out to be positive out of 46 live births (4.35%).[13]

Two seropositive children were delivered vaginally and two had received mixed feeding. In case of two positive children, mothers were not on ART despite recommendations. In case of seropositive ANCs, elective cesarean section is preferred as well as mixed feeding is never recommended.[11] Outcome of child’s sero-status is dependent upon these factors also and inter-dependency of each of these in positive children is further described in Table 3.

**Table 3:** Combined analysis of determinants for all three seropositive children

Child no.	MB Pair	Mode of delivery	Breastfeeding history	Mother on ART
Child 1	Not received	Cesarean section	Mixed feeding	Mother not on ART despite recommendation
Child 2	Not received	Vaginal delivery	Mixed feeding	Mother on ART in accordance with recommendation
Child 3	Not received	Vaginal delivery	Exclusive breastfeeding	Mother not on ART despite recommendation

Presence of one or more risk factor was found in each seropositive child. Role of breastfeeding and mode of delivery in association with sdNVP intervention in the vertical HIV transmission among the uninfected babies may be explored further on a larger cohort of seropositive children.

Given the resources spent after PPTCT services and the role of intrapartum MB pair sdNVP administration, cornerstone of successful program implementation remains to be institutional deliveries of seropositive ANCs. Year-wise analysis of PPTCT services [Table 4] indicates improved rate of institutional deliveries of seropositive ANCs from 53% (2005–’06) to 67.2% (by June 2008). Simultaneously, rate of MB pair coverage in live births has dropped down from 98.3% to 88.9%. Marked improvement is seen in quality of data keeping at ICTCs for the seropositive ANCs.

**Table 4:** Year-wise PPTCT service utilization

Year-wise service delivery and utilization	2005-’06	2006-’07	2007-’08 and till June 2008	Total
Number of seropositive pregnant women detected	117	159	265	541
Institutional deliveries	62	75	178	315
2A. Cesarian section	6	5	27	38
2B. Vaginal delivery	30	44	134	208
2C. Lack of information	26	26	17	69
Number of live births	60	74	171	305
MB pair coverage with ARV prophylaxis	59	70	152	281

## CONCLUSIONS

In order to improve access and reduce various gaps and dropouts, PPTCT services which are feasible in government hospitals should be made available to all ANCs with necessary awareness campaigns especially in rural areas which constitute a major chunk of the population. Presence of gynecologist or doctors thus trained in all such settings is bare minimum.

Continuous capacity building of the ICTC counselor and concerned staff, effective BCC and more involvement of PLWHAs in order to improve quality of services thus become essential.

Every ANC visiting the health facility should go for HIV counseling first and then to the doctor in order to improve the access of PPTCT services. Suitable mechanism may be developed centrally as well as locally.

ANC testing gaps and sdNVP-MB pair, post-test counseling gaps, liaisoning with existent RCH systems and linkages with other services are indicators of the counselor’s strengths or weaknesses. Effective counseling and testing services with promotion of institutional deliveries, interventions can successfully be made available to all seropositive ANCs. Post-test counseling with apropos tracking and follow-up of every seropositive ANC based on her EDD should be ensured with better utilization of ORWs. Maintenance of line-list of such cases may prove immensely beneficial in terms of their institutional deliveries.

Lack of MB pair coverage despite institutional delivery of seropositive ANC is also an area of concern. Drug unavailability in the health setting, absence of adequately knowledgeable staff etc. areas can be dealt with centrally and locally.

Postnatal counseling is a badly neglected area which results in faulty feeding practices that can be improved by combined efforts of counselors, PLWHA and ORWs.

Nil seropositivity in all exposed children who received MB pair shows the efficacy of sdNVP MB pair; however, when it is pursued as the case study mentioned here, the other factors are also pertinent. Although, simple intervention strategies like sdNVP can go a long way in reducing the vertical transmission and pediatric HIV infection significantly in the country.
